# Facilitated detection of social cues conveyed by familiar faces

**DOI:** 10.3389/fnhum.2014.00678

**Published:** 2014-09-02

**Authors:** Matteo Visconti di Oleggio Castello, J. Swaroop Guntupalli, Hua Yang, M. Ida Gobbini

**Affiliations:** ^1^Department of Psychological and Brain Sciences, Dartmouth CollegeHanover, NH, USA; ^2^Department of Medicina Specialistica, Diagnostica e Sperimentale (DIMES), Medical SchoolUniversity of Bologna, Italy

**Keywords:** face perception, familiar face recognition, attention, visual search, eye gaze, head angle, social cognition

## Abstract

Recognition of the identity of familiar faces in conditions with poor visibility or over large changes in head angle, lighting and partial occlusion is far more accurate than recognition of unfamiliar faces in similar conditions. Here we used a visual search paradigm to test if one class of social cues transmitted by faces—direction of another's attention as conveyed by gaze direction and head orientation—is perceived more rapidly in personally familiar faces than in unfamiliar faces. We found a strong effect of familiarity on the detection of these social cues, suggesting that the times to process these signals in familiar faces are markedly faster than the corresponding processing times for unfamiliar faces. In the light of these new data, hypotheses on the organization of the visual system for processing faces are formulated and discussed.

## Introduction

In previous work we have proposed that recognition of familiar faces is based on activation of a distributed network of areas including the theory of mind areas and areas involved in the emotional response (Gobbini et al., 2004; Leibenluft et al., 2004; Gobbini and Haxby, 2006, 2007; Gobbini, 2010). In this manuscript we present new data in the context of a series of psychophysical experiments that focus on visual processing of familiar faces.

We are constantly exposed to faces and face perception is extremely efficient and quick. Even in the context of disrupted visual awareness through various forms of masking and interocular suppression, faces seem to be detected and processed by the visual system more so than other categories of stimuli. For example, upright faces break through interocular suppression one-half second faster than do inverted faces, indicating that the upright facial configuration is processed even when the subject is unaware of the image (Jiang et al., 2007; Yang et al., 2007; Zhou et al., 2010). Social cues such as facial expressions, head direction, and eye gaze direction also appear to be processed when the subject is unaware of the face image, as evidenced by faster breakthrough of interocular suppression by faces with fearful expressions, faces presented in full-frontal view, and faces with eye gaze directed at the viewer (Jiang and He, 2006; Yang et al., 2007; Stein et al., 2011; Gobbini et al., 2013a). Neural response to masked or suppressed faces with fearful expression has been reported in the amygdala suggesting the possibility of a subcortical pathway for fast processing of socially relevant stimuli (Morris et al., 1998; Whalen et al., 1998; Williams et al., 2004; and for review see Tamietto and de Gelder, 2010; but see also Pessoa and Adolphs, 2010; and Valdés-Sosa et al., 2011).

Measurement of saccadic reaction has shown that we can detect a face as fast as 100 ms after stimulus onset (Crouzet et al., 2010). Some research supports the idea that faces, as colors, shapes or orientation might be processed pre-attentively (according to the definition of parallel processing proposed by Treisman and Gelade, 1980), in an automatic way (Hershler and Hochstein, 2005 but see also VanRullen, 2006). Interestingly, the first face-specific evoked potential has been consistently reported at around 170 ms post-stimulus (Bentin et al., 1996; Puce et al., 1999; Eimer and Holmes, 2002) raising the question of which aspect and what level of processing at short latencies (before the N170) is performed to enable rapid face detection.

According to our functional model on face perception (Haxby et al., 2000, 2002) the encoding of the structural aspect of a face that affords recognition of identity is performed by a distinct pathway as compared to the one that is involved with perception of facial movements and, more generally, biological motion (Allison et al., 2000; O'Toole et al., 2002; Winston et al., 2004; Gobbini et al., 2007, 2011; Pitcher et al., 2012). While the ventral temporal pathway, in particular the fusiform gyrus seems to be involved in recognition of the unchangeable aspect of a face, the posterior superior temporal sulcus (pSTS) seems to be involved with perception of the changeable aspects of a face. The STS also seems to be involved in detecting other people's direction of attention. Neurons in the anterior temporal cortex of the monkey are tuned to direction of others' social attention cues, such as head orientation, eye gaze and body movements (Perrett et al., 1985). In humans, fMRI has shown specific regions such as the posterior and anterior superior temporal sulcus, the fusiform gyrus, the medial prefrontal cortex, preferentially engaged by eye gaze and head turns highlighting how dedicated neuronal population are involved in processing relevant social cues (Hoffman and Haxby, 2000; Pageler et al., 2003; Pelphrey et al., 2003; Engell and Haxby, 2007; Schweinberger et al., 2007; Carlin et al., 2012; and for a review Senju and Johnson, 2009).

We have shown that personally familiar faces are detected more efficiently than are faces of strangers in conditions in which attentional resources are reduced and in which faces are rendered subjectively invisible (Gobbini et al., 2013b). Visual search paradigms used by others have reported faster detection of familiar faces in a visual search paradigm (Tong and Nakayama, 1999; see also Deuve et al., 2009) and showed that detecting a specific identity involves a serial search with no pop-out. In Tong and Nakayama (1999), detection of one's own face or a familiar face was faster than detection of unfamiliar faces with a smaller effect of familiarity on search speed that was not significant in one experiment and less than half of the effect on detection speed in a second experiment.

With the present experiment we tested whether social cues, which are supposedly processed by a distinct pathway from that for identity, are detected more efficiently if conveyed by familiar faces. We predicted that the familiarity of a face affects not only the visual representation of invariant aspects for identification, but also the perception of subtle changes that can signal an internal state, such as direction of attention. The extensive expertise with a familiar face might result in efficient processing that is independent of capture of attention. We used a visual search paradigm in which the task is to detect a target with a specified direction of attention—toward or away from the viewer—as conveyed by the gaze direction or head angle of personally familiar or unfamiliar. Importantly, all distractors on target present trials were unfamiliar faces to avoid confounding the effect of faster processing of the target social cue in a familiar face from attentional capture by the familiar face—an effect that would lead to biasing search to check the familiar face containing the target feature earlier than the distractor faces (such a confound muddied the interpretation of results in Buttle and Raymond, 2003). If distractors are familiar faces, a shallower slope for the effect of set size on reaction time (response time vs. set size function, RSF) could be due to faster processing of the familiar face distractors rather than to attentional biasing of a serial search, as was the case in Persike et al. (2013). Thus, in our paradigm an effect of the familiarity of the face with the target feature on the RSF would indicate attentional capture unconfounded by faster processing of distractors. Conversely, an effect of familiarity on target social cue detection independent of an effect on RSF would indicate faster processing in familiar faces independent of attentional capture. Results showed no effect of the familiarity of the target face on the RSF, indicating that the main effect of familiarity on reaction time that was constant across set sizes was due to faster processing of only the target stimulus, not to altered processing of distractors or to an attention-driven bias to process familiar target stimuli earlier in a visual search.

Thus, our results confirm our prediction. Two facial cues for others' direction of attention—gaze direction and head angle—are detected much faster if the faces are personally familiar, corroborating our previous findings on facilitated detection of personally familiar faces under conditions of lack of awareness and reduced attentional resources (Gobbini et al., 2013b). These results suggest that the learned representation involves more than invariant features for identifying familiar individuals but also changeable features for social communication.

## Methods

### Participants

Two sets of four friends (three females, five males) participated in the experiment. As a criterion for familiarity, we chose friends that had extensive interaction with each other for more than a year before the experiment. They were recruited from the Dartmouth College community. Their pictures were taken in different head and gaze orientations to be used as stimuli in the experiment. To ensure that all the stimuli were equal in terms of image quality, we took the pictures in a photo studio with identical lighting and camera placement and settings. Subjects were reimbursed for their participation; all gave written informed consent to use their pictures and to participate in the experiment. The experiment was approved by the local IRB committee.

### Stimuli

For each subject we created three sets of images: target familiar faces (three identities), target unknown faces (three identities), and distractor unknown faces (five identities). Three target unknown individuals were pseudo-randomly sampled from a set of eight identities (four females). Five different identities were used as distractors. Images of the distractor face identities were never used as targets. The pictures of the eight unfamiliar individuals had been previously taken at the University of Vermont with the same lighting, camera placement and settings used for the friends.

Images were cropped, resized to 150 × 150 pixels, and then grayscaled using ImageMagick (version 6.8.7-7 Q16, x86_64, 2013-11-27) on Mac OS X 10.9.2. The average pixel intensity of each image (ranging from 0 to 255) was set to 128 with a standard deviation of 40 using the SHINE toolbox (function *lumMatch*) (Willenbockel et al., 2010) in MATLAB (version 8.1.0.604, R2013a).

### Experimental setup

The experiment was run on an Apple MacPro 1,1, display Apple Cinema HD (23”) set at a resolution of 1280 × 800 pixels with a 60 Hz refresh rate, using Psychtoolbox (version 3.0.8) (Brainard, 1997; Pelli, 1997; Kleiner et al., 2007) in MATLAB (version 7.8.0.347, R2009a).

Before the actual experiment, subjects practiced the task with a set of unrelated images. They sat at a distance of approximately 80 cm from the screen (eyes to screen) in a dimly lit room. The experiment consisted of four different tasks (see below for a detailed description) divided into four blocks. At the beginning of each block, a visual cue indicated the current task. After two blocks, the script invited the subjects to take a break and let the experimenter know they completed the first part of the experiment. After this break, the experimenter ran the script for the second part, and subjects completed the last two blocks. The order of the tasks was randomized.

**Figure 1:**
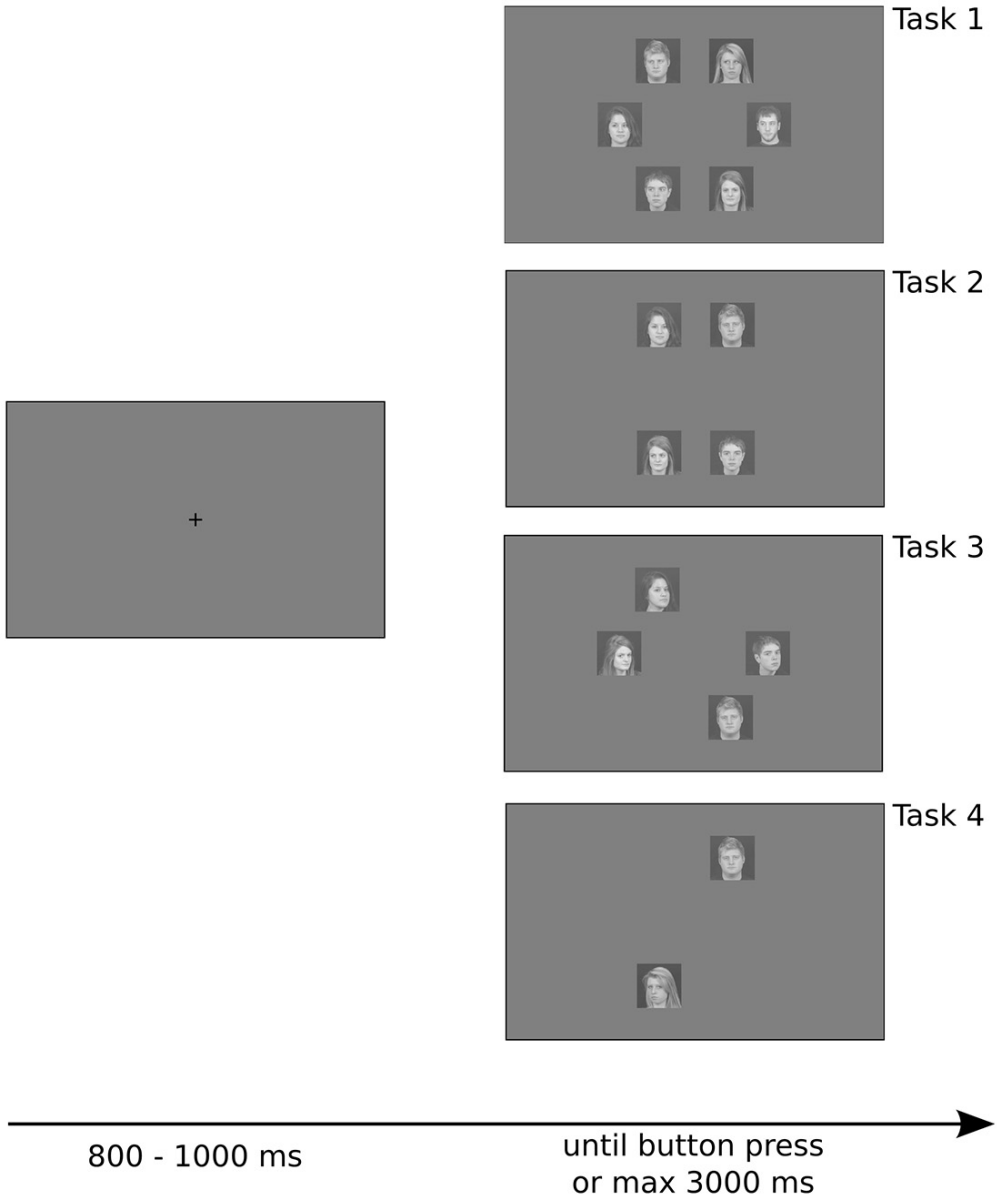
**Example of trials with different number of stimulus array used in the experiment**. Stimuli were positioned on a circle, separated by 60° from each other, making them equidistant from the fixation point and lying on a regular hexagon. Note that for set sizes of two and four there are three possible shapes that the stimuli can create (rotations of 60 and 120° of the shape depicted here), which were randomly chosen from trial to trial. See details in the text.

Stimuli were presented on a gray background (pixel intensity set to 128 for all the pixels), and were positioned approximately 6.89° from the fixation point. Each stimulus had a retinal size of approximately 4.08 × 4.08°. Intertrial intervals were randomly jittered from trial to trial, ranging from 800 to 1000 ms, during which subjects were required to maintain fixation on a black cross in the center of the screen. Stimulus presentation ended with the subject's response or after 3000 ms if no response was made. Subjects were not required to maintain fixation during stimulus presentation (Figure 1).

### Tasks

Subjects were required to detect a target among a different number of distractors (set of 2 or 4 or 6 stimuli), and had to press the left arrow-key (YES) when they found the target, or the right arrow-key (NO) if the target was absent. They heard a beep if they were wrong or if they took too much time to respond (maximum allowed time of 3 s).

The experiment had four tasks. The first two tasks investigated detection of a target with gaze orientation that differed from distractors, controlling for head orientation—all stimuli depicted faces in frontal view. In Task 1 subjects detected a face with gaze directed to the observer among faces with averted gaze. In Task 2 they detected a face with averted gaze among faces with gaze directed to the observer. The other two tasks investigated detection of a target with head orientation that differed from distractors, controlling for gaze orientation—all stimuli depicted faces with gaze directed to the observer. In Task 3 subjects detected a face in full view among faces in profile view (head turned approximately 40°). In Task 4 subjects detected a face in profile view among faces in full view. The order of the tasks was randomized for each participant.

We manipulated the set size (total number of stimuli on the screen: 2, 4, or 6), the familiarity of the target, and the presence of the target. For all set sizes, the stimuli were positioned on a circle with a radius of 250 px (or 6.89° of visual angle) centered on the fixation point, and were positioned on the vertices of a regular hexagon. Thus, all stimuli were equidistant from the fixation point, and the first saccade covered the same distance regardless of the condition. We controlled the position of the stimuli such that the shape they created was always symmetrical with respect to the fixation point (see Figure 1). Thus, the total number of possible shapes was 3, 3, and 1 respectively for set sizes of 2, 4, and 6 (for set sizes of 2 and 4, the other possible shapes are rotations of 60 and 120° of the shapes in Figure 1).

Since we were unable to completely cross the target position and the possible shapes due to time constraints for the experiment, we decided to balance the occurrence of the target in the left and right hemifield, thus avoiding any lateral bias. The shape and the target position were randomly determined for each trial with the constraint that in 50% of the trials the target was on the left side.

The target could be either a familiar or a stranger face. Likewise, on each target absent trial one distractor image was a target face identity (familiar or stranger) with the same gaze and head orientation as the other distractors. Half of target absent trials had a familiar target identity as a distractor, and half had a stranger target identity as a distractor. Thus, the presence of a target identity was not informative on the presence of a target gaze or head orientation.

We also controlled for rightward and leftward orientation of gaze and head angle of targets in Tasks 2 and 4, in which the target had either averted gaze or averted head angle. The orientation of the targets was balanced to the left and right. In Tasks 1 and 3 the orientation of the distractors was similarly balanced. For each trial, all distractors were oriented to one side. Half of the trials had all distractors oriented to the left, and the other half had all distractors oriented to the right.

For each task we presented each target identity two times for each set size, target present or absent, and right- or leftward orientation condition, thus yielding 144 trials per task (Number of target identities × 2 × Set size × Presence of target × Orientation = 6 × 2 × 3 × 2 × 2 = 144). The trial order was randomized.

## Results

We analyzed reaction times for target present and target absent trials separately. Table 1 shows the Reaction Times (RTs) in ms and Table 2 shows mean *d*' values and SE for each task and each condition.

**Table 1:** **Mean RTs [ms] for each condition and each task, correct responses only**.

	**Target present**			**Target absent**		
	**2**	**4**	**6**	**2**	**4**	**6**
**TASK 1: LOOK FOR THE FACE LOOKING AT YOU**						
Familiar	841.13	989.91	1227.41	942.00	1339.49	1693.31
Stranger	911.22	1155.24	1293.02	934.42	1371.39	1767.12
**TASK 2: LOOK FOR THE FACE LOOKING AWAY FROM YOU**						
Familiar	828.95	1034.80	1139.26	924.90	1328.14	1670.04
Stranger	911.12	1135.08	1260.31	920.76	1328.76	1662.29
**TASK 3: LOOK FOR THE FACE TURNED AT YOU**						
Familiar	755.27	912.25	1021.39	871.81	1157.21	1487.33
Stranger	784.89	1010.57	1210.31	855.82	1180.30	1524.27
**TASK 4: LOOK FOR THE FACE TURNED AWAY FROM YOU**						
Familiar	748.21	909.23	1048.50	853.01	1060.19	1322.26
Stranger	783.78	969.46	1051.81	807.67	1118.32	1347.78

**Table 2:** **Mean *d*' values and SE (*N* = 8, in parenthesis) for each task and condition**.

	**2**	**4**	**6**
**TASK 1: LOOK FOR THE FACE LOOKING AT YOU**			
Familiar	3.25 (0.15)	3.35 (0.12)	3.40 (0.09)
Stranger	3.11 (0.14)	2.80 (0.20)	2.66 (0.23)
**TASK 2: LOOK FOR THE FACE LOOKING AWAY FROM YOU**			
Familiar	3.31 (0.22)	2.78 (0.24)	2.85 (0.33)
Stranger	3.14 (0.12)	2.73 (0.22)	2.72 (0.21)
**TASK 3: LOOK FOR THE FACE TURNED AT YOU**			
Familiar	3.32 (0.10)	3.25 (0.11)	3.06 (0.26)
Stranger	3.10 (0.18)	2.81 (0.22)	2.81 (0.21)
**TASK 4: LOOK FOR THE FACE TURNED AWAY FROM YOU**			
Familiar	3.35 (0.12)	3.21 (0.22)	3.10 (0.14)
Stranger	3.17 (0.20)	3.10 (0.19)	3.11 (0.18)

Data were analyzed in R (version 3.0.2, R Core Team, 2013) using a Linear Mixed-Effect Model on RTs and *d*' values, as implemented in the package *lme4* (version 1.0-6, Bates et al., 2014). The model was then fitted with Maximum-Likelihood estimation. To find the best fitting model, different models were evaluated according to the AIC (Akaike Information Criterion), and tested by means of a log-likelihood ratio test (Baayen et al., 2008). Once the best model was found, interaction or main fixed effects of this model were also evaluated with a log-likelihood ratio test (Baayen et al., 2008).

Reliability of parameter estimates for main fixed effects and contrasts were evaluated through parametric bootstrapping (10,000 replicates), and then computing 95% basic bootstrap confidence intervals (bCI). Effect sizes for familiarity and 95% bCa confidence intervals (10,000 repetitions) shown in Tables 3, 4 were computed using the package *bootES* (version 1.01, Kirby and Gerlanc, 2013).

**Table 3:** **Unstandardized effect size [ms] of familiarity for the Target Present condition and Cohen's d effect size of familiarity across set sizes in the four tasks (bCa bias-corrected and accelerated confidence intervals, computed with 10,000 repetitions)**.

**Target present: Familiar vs. Stranger**			
**Set size**	**Effect size [ms]**	**95% bCa**	**SE**
**TASK 1: LOOK FOR THE FACE LOOKING AT YOU**			
2	−72.67	[−149.22, −24.04]	32.85
4	−173.35	[−280.29, −88.94]	51.61
6	−87.99	[−192.67, 21.81]	59.17
Overall	−111.34	[−168.95, −58.50]	28.64
Cohen's d	−0.79	[−1.20, −0.33]	0.23
**TASK 2: LOOK FOR THE FACE LOOKING AWAY FROM YOU**			
2	−82.45	[−141.86, −36.16]	28.47
4	−121.38	[−202.10, −61.29]	37.13
6	−113.80	[−239.69, 46.90]	76.08
Overall	−105.87	[−158.27, −48.29]	28.66
Cohen's d	−0.75	[−1.30, −0.22]	0.29
**TASK 3: LOOK FOR THE FACE TURNED AT YOU**			
2	−31.87	[−91.10, 16.68]	29.48
4	−89.03	[−167.49, −27.73]	37.82
6	−166.21	[−225.29, −96.00]	34.94
Overall	−95.70	[−139.92, −54.96]	22.11
Cohen's d	−0.88	[−1.30, −0.47]	0.22
**TASK 4: LOOK FOR THE FACE TURNED AWAY FROM YOU**			
2	−37.60	[−135.78, 5.36]	33.59
4	−66.19	[−177.44, 24.41]	55.22
6	2.30	[−110.56, 95.04]	55.64
Overall	−33.83	[−92.40, 16.03]	27.79
Cohen's d	−0.25	[−0.66, 0.17]	0.21

**Table 4:** **Unstandardized effect size [ms] of familiarity for the Target Absent conditions and Cohen's d effect size of familiarity across set sizes in the four tasks (bCa bias-corrected and accelerated confidence intervals, computed with 10,000 repetitions)**.

**Target absent: Familiar vs. Stranger**			
**Set Size**	**Effect Size [ms]**	**95% bCa**	**SE**
**TASK 1: LOOK FOR THE FACE LOOKING AT YOU**			
2	8.06	[−47.17, 32.78]	19.31
4	−26.55	[−91.56, 33.75]	34.63
6	−67.91	[−141.79, −22.10]	30.91
Overall	−28.80	[−65.77, 1.13]	17.27
Cohen's d	−0.34	[−0.70, 0.06]	0.19
**TASK 2: LOOK FOR THE FACE LOOKING AWAY FROM YOU**			
2	3.41	[−80.10, 71.01]	41.31
4	−2.36	[−47.92, 44.61]	24.95
6	3.28	[−143.78, 68.65]	51.37
Overall	1.44	[−51.11, 37.60]	22.46
Cohen's d	0.01	[−0.40, 0.47]	0.22
**TASK 3: LOOK FOR THE FACE TURNED AT YOU**			
2	18.25	[−85.08, 80.72]	42.90
4	−22.01	[−130.04, 57.16]	49.99
6	−41.30	[−144.60, 53.13]	53.69
Overall	−15.02	[−74.48, 33.38]	27.56
Cohen's d	−0.11	[−0.52, 0.32]	0.21
**TASK 4: LOOK FOR THE FACE TURNED AWAY FROM YOU**			
2	45.43	[−0.51, 130.58]	33.45
4	−51.51	[−135.82, 13.64]	39.85
6	−24.39	[−122.19, 69.47]	52.09
Overall	−10.15	[−58.77, 36.49]	24.95
Cohen's d	−0.08	[−0.49, 0.35]	0.21

### Target present

We first created a general model entering main effects of task, set size, and familiarity of the target, and the interaction between set size and familiarity; subjects and target items were entered as random effects with random intercepts and random slopes for familiarity. Then we removed random slopes for familiarity (one at a time) to test whether a parsimonious model could be found. Indeed, we found that removing random slopes for both random effects decreased the AIC, while the X^2^ log-likelihood ratio tests were not significant.

The RSF for familiar and unfamiliar targets were not significantly different, as indicated by a non-significant interaction between familiarity and set size (X^2^_(1)_ = 1.28, *p* = 0.26). Consequently, we further simplified the model by removing this interaction effect. Thus, this yielded the best model in terms of AIC with task, set size, and familiarity as main fixed effects, and subjects and target items as random effects with random intercepts.

**Figure 2:**
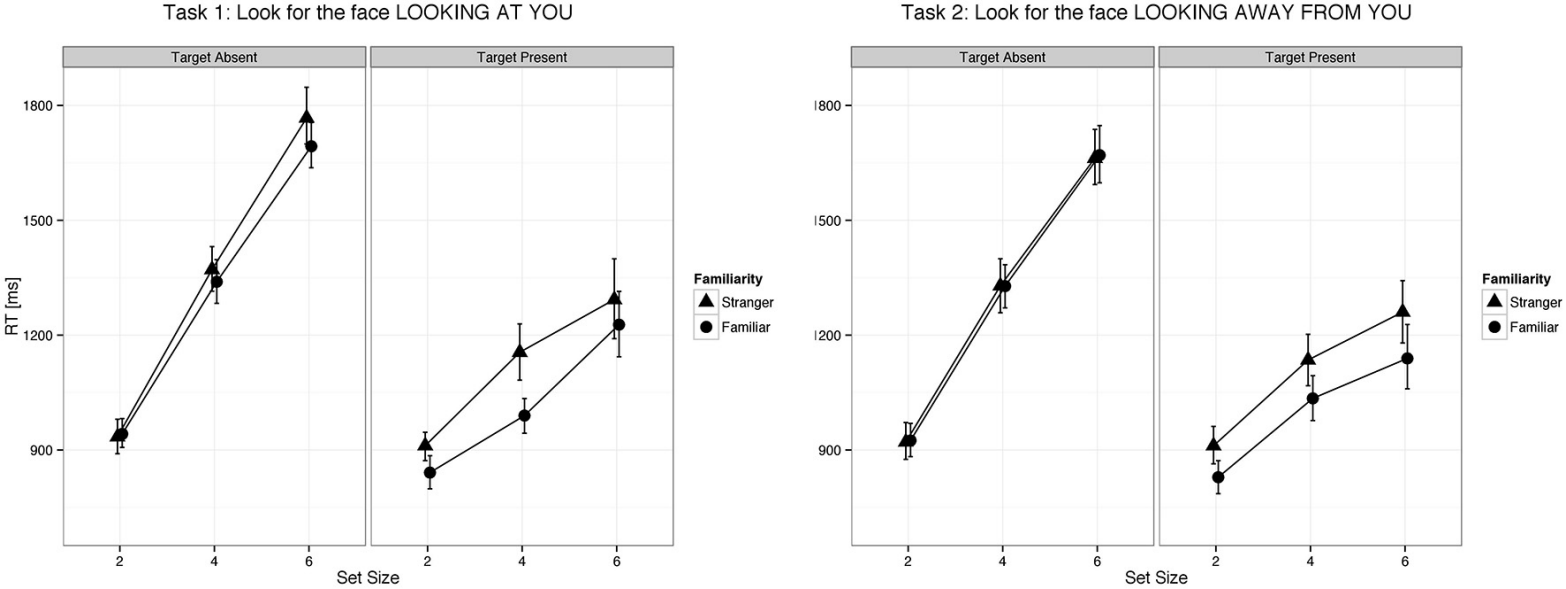
**Eye gaze was detected faster in familiar faces than in unfamiliar faces both when it was directed to the viewer and when it was averted**. Error bars represent 95% bootstrapped confidence intervals.

**Figure 3:**
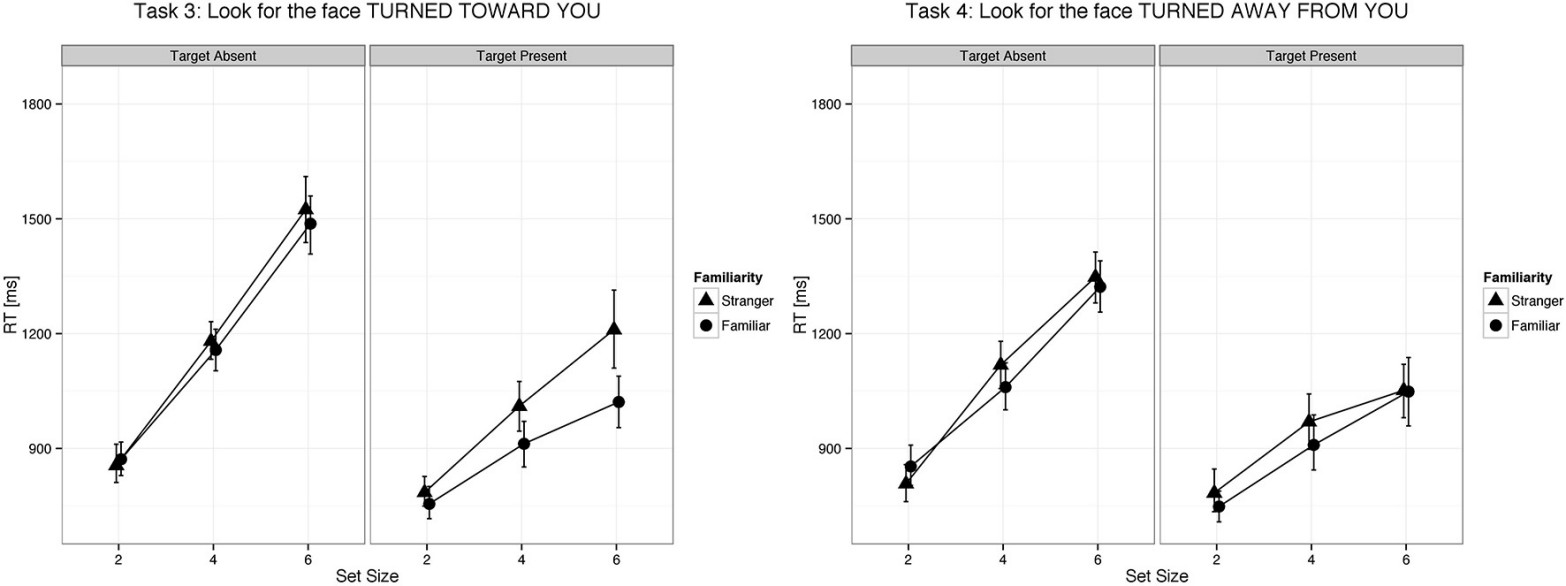
**Changes in head position of familiar faces were detected faster as compared to changes in head position of unfamiliar faces**. Error bars represent 95% bootstrapped confidence intervals.

We found a main effect of familiarity (X^2^_(1)_ = 21.07, *p* < 0.0001, parameter estimate = −83.8 ms, 95% bCI: [−115.7, −52.1]), set size (X^2^_(1)_ = 385.35, *p* < 0.0001, parameter estimate = 168.6 ms, bCI: [152.4, 185.1]), and task (X^2^_(3)_ = 73.94, *p* < 0.0001). The strong effect of set size on target present trials for all tasks indicates that visual search for gaze direction and head angle is serial with no evidence for parallel search or pop-out. Mean slope for the RSF on target present trials for gaze detection was 91 ms/item for gaze direction and 77 ms/item for head angle. Mean difference time for detection of target social cues in familiar and unfamiliar faces was 109 ms for gaze direction and 65 ms for head angle. We found a statistical difference between the two tasks (Gaze vs. Head, parameter estimate = 62.97 ms, bCI: [49.2, 76.7]), but no difference between Task 1 and Task 2 (parameter estimate = 9.18 ms, bCI: [−11.5, 30.1]) nor between Task 3 and Task 4 (parameter estimate = 15.57 ms, bCI: [−5.1, 36.1]). For an overview of all results in the Target Present conditions see Tables 1–3, 5, and Figures 2, 3.

**Table 5:** **RSF slope estimates for Target Present and Target Absent conditions for the four tasks and for Familiar/Stranger targets (bCa bias-corrected and accelerated confidence intervals, computed with 10,000 repetitions)**.

**Task**	**Familiar**		**Stranger**	
	**Slope [ms/item]**	**95% bCa**	**Slope [ms/item]**	**95% bCa**
**TARGET PRESENT**				
Look AT	96.92	[78.14, 114.83]	100.75	[63.68, 128.79]
Look AWAY	79.20	[63.29, 94.29]	87.04	[59.44, 107.46]
Turn AT	66.72	[45.89, 96.26]	100.30	[74.13, 134.84]
Turn AWAY	76.25	[52.17, 97.77]	66.28	[41.78, 88.46]
**TARGET ABSENT**				
Look AT	189.23	[169.36, 212.27]	208.23	[180.41, 227.53]
Look AWAY	185.66	[153.98, 218.36]	185.70	[151.22, 215.19]
Turn AT	152.22	[119.09, 182.93]	167.11	[132.52, 204.42]
Turn AWAY	117.93	[93.66, 149.14]	135.39	[112.93, 157.53]

### Target absent

We ran the same analysis for target absent and found that the best model was again with task, set size, and familiarity as main fixed effects, and subjects and target items as random effects with random intercepts. All interactions (two-way and three-way) were not significant.

We found a main effect of task (X^2^_(3)_ = 215.88, *p* < 0.0001) and set size (X^2^_(1)_ = 1443.3, *p* < 0.0001, parameter estimate = 335.5 ms, bCI: [320.9, 350.0]), but not for familiarity (X^2^_(1)_ = 1.3, *p* = 0.26, parameter estimate = −15.1 ms, bCI: [−41.9, 11.2]). Mean slope for the RSF on target absent trials for gaze detection was 192 ms/item for gaze direction and 143 ms/item for head angle. A contrast of tasks showed that the first two tasks were statistically different from the last two (Gaze vs. Head, parameter estimate = 95.0 ms, bCI: [82.6, 107.0]), and that Task 3 was statistically different from Task 4 (Detect Full View vs. Detect Profile View, parameter estimate = 47.2 ms, bCI: [29.6, 65.1]), but Task 1 was not statistically different from Task 2 (Detect Direct Gaze vs. Detect Averted Gaze, parameter estimate = 17.6 ms, bCI: [−0.1, 35.6]). For an overview of all results in the Target Absent conditions see Tables 1, 2, 4, 5, and Figures 2, 3.

### *d*' values

Since many subjects had False Alarm rates of 0, we computed the Hit and FA ratios by adding 0.5 and dividing by N + 1, thus scaling the ratios to avoid extremes. To analyze *d*' values, we used the same analyses (Linear Mixed-Effect Models) as described above. We found that the best model was with task, set size, and familiarity as main fixed effects, and subjects as random effects with random intercepts. All interactions (two-way and three-way) were not significant.

We found a main effect of set size (X^2^_(1)_ = 10.26, *p* = 0.0014, parameter estimate = −0.1284 [−0.2074, −0.0509]), familiarity (X^2^_(1)_ = 14.32, *p* = 0.0002, parameter estimate = 0.2490 [0.1258, 0.3769]), and task (X^2^_(3)_ = 7.83, *p* = 0.0497). The contrasts for task we specified before were not statistically significant for the *d*' values: Gaze vs. Head angle, parameter estimate = −0.0538 [−0.3510, 0.0060]; Task 1 vs. Task 2, parameter estimate = 0.0872 [−0.2134, 0.1430]; Task 3 vs. Task 4, parameter estimate = −0.0570 [−0.1081, 0.2547] (see Table 2 for the mean *d*' values and SE for each condition and each task).

## Discussion

Face perception is arguably one of the most developed visual skills in humans. Faces are detected more readily than other objects (Crouzet et al., 2010). Familiar face perception is especially sensitive and efficient and is dramatically better than unfamiliar face perception (Jenkins and Burton, 2011). Here we show that one class of social cues transmitted by faces—perception of the direction of another's attention—is detected much more rapidly in familiar faces than in unfamiliar faces. In previous work, we have shown that personally familiar faces, as compared to faces of strangers, are detected more readily in conditions with reduced attentional resources and even without awareness (Gobbini et al., 2013b). With the experiments reported in the present manuscript, we extend this line of research to show that the increased efficiency afforded by familiarity includes not only simple detection but also the perception of socially-relevant cues.

We used a visual search paradigm to test the effect of face familiarity on the detection of a target with a different gaze or head orientation. We found that the familiarity of the face with the target feature had a strong effect on detection time but no effect on RSF slopes—in other words, a facilitation of social cue detection that was constant across set sizes. This result indicates that the social cue was detected much faster in familiar than unfamiliar faces and that attentional capture—a bias to process the familiar faces earlier in a serial visual search—did not play a significant role, as such an effect would be reflected in a flatter RSF.

As expected we found that increasing the number of distractors made the task harder as evidenced by increased reaction times and decreased *d*' values. Moreover, as expected, we found that detecting a target head orientation was faster than detecting a target gaze direction, albeit with no difference in accuracy. This effect could be due to the fact that head orientation differences are evident in larger changes in the visual stimulus than are gaze direction differences, thus making the visual search easier.

Our results clearly show that detection of target gaze directions and head angles involves a serial visual search with no indication of parallel processing or pop-out. Detection times on target present trials showed a strong effect of set size. This finding is consistent with those of Tong and Nakayama (1999) who found that detection of a target individual (self or a stranger) among distractor faces involved a serial search. Pop-out for simple face detection among non-face distractors was shown in one report using large set sizes (Hershler and Hochstein, 2005) but appears to be due to low level visual features, namely the amplitude spectrum of spatial frequencies (VanRullen, 2006).

Images of familiar and unfamiliar faces were carefully matched. All pictures were made with the same lighting and photographic equipment in a studio setting. Mean luminance and contrast were the same for all stimuli. Thus, spurious low-level differences cannot account for performance differences between the detection of familiar and stranger targets. Indeed, we found a large main effect of familiarity for both the speed and accuracy of target detection.

The slope of the RSF is an indication of how much time is required to check each stimulus for the target feature. Target absent trials require checking all stimuli for the target feature, resulting in RSF slopes that are twice as steep as those for target present trials on which visual search terminates with detection of the target feature. Processing each distractor for gaze orientation, as indicated by the RSF slope on target absent trials, required on average 192 ms, and processing each distractor for head angle required 143 ms. In this context, the effect of familiarity on gaze orientation and head angle tasks (109 ms and 65 ms, respectively) suggests that the times to process these signals in familiar faces are markedly faster than the corresponding processing times for unfamiliar faces.

Familiar faces also may attract attention, biasing visual search to process familiar faces earlier than unfamiliar faces, an effect that also could cause faster detection of social cues in familiar faces. Such an effect, however, would make the RSF slope flatter for familiar target trials than for unfamiliar target trials, an effect that was not significant in the current study. In Tong and Nakayama (1999), the RSF slope was slightly flatter for finding one's own face than for finding an unfamiliar face target in a visual search task. This effect was not significant in their first experiment, with an RSF slope difference of 15 ms/item, and was significant in the second experiment, with an RSF slope difference of 23 ms/item. Estimate of the equivalent effect in our data, based on target present trials as in Tong and Nakayama (1999), was 10 ms/item and not significant. When we include this non-significant effect in a model that accounts for the difference in detection times with both cue processing and RSF slope differences, the facilitation of detection by familiarity is still due mostly to a faster processing of the social cue rather than to looking at familiar faces earlier. The more parsimonious explanation that better fits our data, therefore, is that the target social cue—gaze angle and head direction—is examined in each stimulus in the search array, that this process is serial, that a familiar face is no more likely than an unfamiliar face to be examined earlier in the serial search, and that the social cue is processed more quickly if the face is familiar.

We also found that responding “no” on target absent trials was slowed by 20–40 ms if the distractors all had attention directed away from the viewer, as indicated either by averted gaze or averted head angle. Perceived gaze and head orientation represent strong signals for reallocating attention in humans, and the attentional shift to the side elicited when someone else stares or turns their head away from us appears to be automatic (Friesen and Kingstone, 1998; Frischen et al., 2007). This automatic diversion of attention may be the underlying cause for slower response times on target absent trials when distractor face images had averted gaze or head angle. To summarize, not only are familiar faces detected faster than are faces of strangers (Tong and Nakayama, 1999; Deuve et al., 2009; Ramon et al., 2011; Gobbini et al., 2013b) but also cues that represent strong social signals (Perrett et al., 1985; Senju and Johnson, 2009; Stein et al., 2011; Gobbini et al., 2013a)—eye gaze and head direction—are detected much more rapidly if they are perceived in a familiar face.

We spend a great amount of time at looking at faces of immediate family and close friends that become intimately familiar over repeated exposure and social interaction extending over years. This slow and prolonged exposure can contribute to the development of a more stable representation of the visual appearance of a familiar face. Personally familiar faces, in contrast to the faces of strangers, are detected faster and recognized with great efficiency in conditions of poor visibility and over large changes in a head angle, lighting, partial occlusion, and age (Burton et al., 1999; O'Toole et al., 2006; Johnston and Edmonds, 2009; Burton and Jenkins, 2011). Personally familiar faces are among the most highly-learned and salient visual stimuli for humans and are associated with changes in the representation of both the visual appearance and associated person knowledge, affording highly efficient and robust recognition. By contrast, recognition of unfamiliar faces—identifying a target unfamiliar face among other faces—is surprisingly inaccurate (Burton et al., 1999; O'Toole et al., 2006; Burton and Jenkins, 2011). Whereas the performance of machine vision systems for face recognition is equivalent to human performance for unfamiliar face recognition, human performance for familiar face recognition is much better (Jenkins and Burton, 2011; O'Toole et al., 2011). Understanding the perceptual and neural mechanisms underlying this remarkable performance is of great interest for understanding how neural systems become highly efficient for highly salient stimuli and for designing better machine vision systems. The relative roles played by detectors for fragmentary or holistic visual features and by top-down influences of semantic information in the facilitation of familiar face processing are unknown. Face detection and perception of the direction of another's attention, however, appear to be extremely fast, efficient, and independent of attentional resources and even awareness (Jiang et al., 2007; Crouzet and Thorpe, 2011; Gobbini et al., 2013a), suggesting that top-down influences of semantic information may play a minor role and that facilitation of familiar face processing may be due mostly to the development of detectors of fragmentary or holistic visual features that are specific to familiar individuals.

A distributed system for face perception has been described in humans (Haxby et al., 2000, 2002; Ishai et al., 2005; Gobbini and Haxby, 2007; Haxby and Gobbini, 2011) and monkeys (Tsao et al., 2008; Freiwald and Tsao, 2010). In humans the system includes visual cortical areas that are involved in perception of invariant visual attributes diagnostic of identity and perception of changeable aspects for facial expression and speech (the “core system”) and additional areas involved in representation of information associated with faces, such as person knowledge, emotion, and spatial attention (the “extended system”) (Haxby et al., 2000, 2002; Ishai et al., 2005; Gobbini and Haxby, 2007; Taylor et al., 2009; Natu and O'Toole, 2011; Bobes et al., 2013). Repeated exposure to faces might result in natural and protracted learning that tunes this hierarchical and distributed system at all levels to afford efficient and robust detection and identification of these faces. This could be due to development of representations of the visual appearance across many different changes in head angle, lighting, expression, and partial occlusion. The integration of multiple representations into a general representation of an individual could help build a system that is stable, robust, and efficient (Bruce, 1994; Burton et al., 2011). Neurophysiological data from monkeys suggest that a view-independent representation of faces is achieved through a series of processing steps from posterior toward more anterior face responsive patches in the temporal cortex that exhibit population responses tuned to head angle more posteriorly (MF/ML) and to head-angle invariant face identity more anteriorly (AM) (Freiwald and Tsao, 2010). In humans, face areas in the core system are tuned differentially to face parts (the occipital face area, OFA), invariant aspects that support recognition of identity (the fusiform face area, FFA) and changeable aspects such as facial expression, eye gaze, and speech movements (the pSTS). In addition, human face areas have been described in anterior temporal and inferior frontal cortices (the ATFA and IFFA) that may play a critical role in identification (Rajimehr et al., 2009; Kriegeskorte et al., 2007; Natu et al., 2010; Nestor et al., 2011; Kietzmann et al., 2012; Anzellotti and Caramazza, 2014; Anzellotti et al., 2014).

Classical cognitive models on face perception and recognition posit that visual recognition necessarily precedes access to person knowledge (Bruce and Young, 1986). Evoked potential studies have shown that the first face-specific response to a face, the N170, is not modulated by familiarity (Bentin et al., 1999; Puce et al., 1999; Eimer, 2000; Paller et al., 2000; Abdel Rahman, 2011 but see also Caharel et al., 2011). Instead, modulation of the response by familiarity appears at later latencies (greater than 250 ms) (Eimer, 2000; Schweinberger et al., 2004; Tanaka et al., 2006). Whereas early face-specific evoked potentials are recorded in posterior temporal locations, the later potentials that are modulated by familiarity are recorded in temporal, frontal and parietal locations (Bentin et al., 1999; Puce et al., 1999; Eimer, 2000; Tanaka et al., 2006). Faster detection without awareness of personally familiar faces as compared to faces of strangers suggest that early face processing that precedes explicit recognition may be facilitated for personally familiar faces (Gobbini et al., 2013b). Models of object perception hypothesize that the recognition of objects despite pronounced changes in appearance is due to a multistep sequence of processing, characterized by stages in which stimulus features of increasing complexity are analyzed and combined until a representation, invariant to visual transformation is achieved in the inferior temporal cortex (Ullman et al., 2002; Riesenhuber and Poggio, 2002; Serre et al., 2007; DiCarlo et al., 2012; but see also Kravitz et al., 2013).

Psychophysical studies have shown that faces can be detected very rapidly, with the earliest reliable saccades to faces at 100–110 ms (Crouzet et al., 2010; Crouzet and Thorpe, 2011). Face specific patterns of neural activity can be detected as early as 100 ms with EEG using multivariate pattern analysis (Cauchoix et al., 2014). These very rapid responses to faces may be due to low-level visual features that are more frequent in faces (Tanskanen et al., 2005). For example, Honey et al. (2008) and Crouzet and Thorpe (2011) demonstrated the importance of specific spatial frequency amplitudes underlying ultra-fast face detection. Specific properties of faces, such as eye gaze direction, head angle and personal familiarity, differentially facilitate detection even without awareness (Stein et al., 2011; Gobbini et al., 2013a,b). These findings raise the question of how such fast and preconscious processing can be achieved—through a subcortical system (for a review see Tamietto and de Gelder, 2010 but see also Pessoa and Adolphs, 2010) or through a cortical route with a fast feed-forward integration of information (VanRullen and Thorpe, 2001) and activation of the distributed network in the fronto-parietal areas for retrieval of person knowledge. Highly-learned representations of personally familiar faces may also include detectors for visual features—face fragments or more holistic configurations—that are diagnostic for familiar individuals (Butler et al., 2010). The facilitation of familiar face processing that appears to be at least partially independent of attentional resources and awareness may be due to activation of such learned diagnostic feature detectors. The results presented here suggest that these detectors also may be specific for features that carry social signals, such as eye gaze direction, head orientation, and expression.

A largely unexplored mechanism in the expertise for familiar faces involves detectors for diagnostic facial features in early visual cortex. Petro et al. (2013) have shown facial attributes such as gender and expression can be decoded, using multivariate pattern analysis (MVPA), in V1 cortical patches. Diagnostic features specific to familiar faces might be learned through experience and might afford “pre-recognition” detection, namely facilitated detection without an explicit recognition of the identity of highly familiar faces. Instead, explicit recognition of a highly familiar face may require top-down processing from neural systems that are involved in retrieval of person knowledge and in the emotional response, and this top-down input could serve to tune and amplify the visual representation of personally familiar faces (Gobbini and Haxby, 2007; Gobbini, 2010).

In this manuscript we have presented new evidence for facilitated processing of personally familiar faces. We have highlighted the importance of testing the human system for familiar face detection and recognition. Experiments using familiar faces as stimuli can offer insight on the organization of the neural systems for recognition of highly familiar objects, can help improve software for face recognition and can shed further light on practical issues such as flaws in eye witness reports. Our expertise with face recognition seems to be most developed for familiar faces, and unfamiliar face recognition is disappointing. Our expertise with familiar faces could be due to the integrated functioning of the distributed neural system for face perception at multiple levels (Haxby et al., 2000, 2001; Gobbini and Haxby, 2007; Haxby and Gobbini, 2011). The extended system components for the representation of person knowledge may interact with the representation of the visual appearance to stabilize and strengthen the representation of visual features that are diagnostic of the identity and facial gestures of familiar individuals. The development of a robust representation of the visual appearances of familiar individuals affords detection even in conditions with poor visibility (O'Toole et al., 2006; Burton and Jenkins, 2011). Activation of these simple features might facilitate detection preceding explicit recognition and facilitate processing of social signals. Understanding how learning tunes integrated processing of personally familiar faces in the hierarchical system for face perception may serve as a model for how learning tunes neural systems for recognition of other highly salient stimuli, such as gestures and actions, personal objects and places, or voices and written words.

## Author contributions

M. Ida Gobbini conceived the idea; M. Ida Gobbini, J. Swaroop Guntupalli and Matteo Visconti di Oleggio Castello designed the experiment; J. Swaroop Guntupalli and Hua Yang collected and analyzed the data of a pilot study; Matteo Visconti di Oleggio Castello collected and analyzed the data for the final experiment; M. Ida Gobbini and Matteo Visconti di Oleggio Castello wrote the manuscript; Hua Yang and J. Swaroop Guntupalli provided critical inputs to the final version of the manuscript.

### Conflict of interest statement

The authors declare that the research was conducted in the absence of any commercial or financial relationships that could be construed as a potential conflict of interest.
